# Comparative analysis of resource utilization in integrative anthroposophic and all German pediatric inpatient departments

**DOI:** 10.1186/s12913-020-05782-6

**Published:** 2020-10-12

**Authors:** Katharina Fetz, Alfred Längler, Melanie Schwermer, Clara Carvalho-Hilje, Jan Vagedes, Tycho Jan Zuzak, Thomas Ostermann

**Affiliations:** 1 Department of Psychology, Chair of Research Methodology and Statistics in Psychology, Witten/Herdecke University, Alfred-Herrhausen-Straße 50, 58448 Witten, Germany; 2 Department of Pediatrics, Gemeinschaftskrankenhaus Herdecke, Gerhard-Kienle-Weg 4, 58313 Herdecke, Germany; 3 Professorship for Integrative Pediatrics, Institute for Integrative Medicine, Witten/Herdecke University, Alfred-Herrhausen-Straße 50, 58448 Witten, Germany; 4 ARCIM Academic Research in Complementary and Integrative Medicine, Filderstadt, Germany; 5 Department of Neonatology, University Hospital Tuebingen, Calwerstraße 7, 72076 Tübingen, Germany; 6 Department of Pediatric Oncology and Hematology, University Hospital Essen, Hufelandstr.55, 45147 Essen, Germany

**Keywords:** Integrative medicine, Pediatrics, Children, Anthroposophic medicine, Resource utilization, Cost analysis, DRG, MDC

## Abstract

**Background:**

Integrative Medicine (IM) combines conventional and complementary therapies. It aims to address biological, psychological, social, spiritual and environmental aspects of patients’ health. During the past 20 years, the use and request of IM in children and adults has grown.

Anthroposophic Medicine (AM) is an IM approach frequently used in children in Germany. From both public health and health economic perspectives, it is relevant to investigate whether there are differences in the resource utilization between integrative pediatric departments (IPD) and the entirety of all pediatric departments.

**Methods:**

Standard ward documentation data from all German integrative anthroposophic pediatric departments (2005–2016; *N* = 29,956) is investigated and systematically compared to data of the entirety of all pediatric departments in Germany derived from the Institute for the Hospital Reimbursement System (2005–2016, *N* = 8,645,173). The analyses focus on: length of stay, Diagnosis Related Groups (DRG), Major Diagnosis Categories (MDC), and effective Case Mix Index (CMI).

**Results:**

The length of stay in the IPD (M = 5.38 ± 7.31) was significantly shorter than the DRG defined length of stay (M = 5.8 ± 4.71; *p* < .001; d = − 0.07) and did not exceed or undercut the DRG covered length of stay. Compared to the entirety of all pediatric departments (M = 4.74 ± 6.23) the length of stay was significantly longer in the in the IPD (*p* <. 001; d = 0.12). The effective CMI in IPD and all pediatric departments were identical (M = 0.76). The frequencies of DRG and MDC differed between IPD and all pediatric departments, with higher frequencies of DRGs and MDCs associated with chronic and severe illnesses in the IPD.

**Conclusions:**

Treatment within integrative anthroposophic pediatric departments fits well in terms of the DRG defined conditions concerning length of stay, even though integrative pediatric patients has an increased length of stay of averagely 1 day, which is most likely associated to time consuming, complex integrative treatment approaches and to a certain extend to higher amount of chronic and severe diseases.

## Background

Integrative Medicine (IM) is “healing-oriented medicine that takes account of the whole person, including all aspects of lifestyle. It emphasizes the therapeutic relationship between practitioner and patient, is informed by evidence, and makes use of all appropriate therapies” [1]. It considers biological, psychological, social, spiritual, and environmental aspects of health [2]. It is not “a discipline, a group of disorders, or a method of treatment, but an approach, a way of thinking”, it “encourages clinicians and researchers to consider more than one system at a time” and “provides a framework for understanding complex and dynamic challenges” of the human organism [3]. IM is practiced worldwide and varies in its special approaches depending on cultural and national factors [4]. Therefore, IM is of particular interest from both, a public health and health economic perspective.

During the last 20 years, the implementation of integrative approaches for children has grown [2, 5–15] worldwide: IM is used in pediatrics in the USA [9, 11, 16–19], in Canada [20] and in Europe [5, 8, 21–25] in private practices, outpatient- and inpatient-departments [23]. Current literature suggests that 30–50% of parents of children with acute or chronic diseases use IM for their children [26–29], while it seems to be used more frequently for children with chronic diseases in the US [16, 30–35]. IM’s use for children is associated with disease severity and whether parents use IM themselves [7, 36]. IM is established in academic pediatrics and is acknowledged as an important subspecialty to address children’s needs [2].

An IM approach with particular relevance in Europe and Germany is Anthroposophic Medicine (AM). It is a multimodal treatment system founded by Rudolf Steiner and Ita Wegmann in the early 1920s [37] and includes complementary pharmacotherapy, medicinal baths, rhythmical massages, compresses, and embrocation (rhythmic massages with etheric oils [38]), as well as art therapy, eurythmy, speech therapies, music therapy [39], and light/ color therapy. Consensus-based guidelines for the anthroposophic therapies for children suffering from general pediatric diseases, such as acute gastroenteritis [40] and bronchitis [41] have already been published.

In Europe, AM is integrated into conventional medical services and practiced in inpatient and outpatient settings. There are two anthroposophic hospitals in Germany that offer integrative treatment for children in distinct pediatric departments: The Gemeinschaftskrankenhaus Herdecke (community hospital) near Dortmund and the Filderklinik in Filderstadt near Stuttgart [23, 42]. A recent study of our working group found a large catchment area for these hospitals all over Germany and that parents are willing to travel further distance to get specialized integrative anthroposophic medical care for children with severe and chronic diseases [43].

However, little is known about the impact of integrative anthroposophic pediatric treatment on resource utilization as one element of economic analysis. Considering the growing costs of health care, a better understanding of resource utilization is indispensable to provide clinically effective and financially responsible treatment. Especially within the integrative field, there is a need for research evaluating the resource utilization and benefits for patients and the health care system [41, 42]. The evaluation of resource utilization parameters thus may provide valuable information that can be considered when seeking to optimize integrative strategies in order to lower health care costs and to license and scope health care investment decisions [44].

Until today there is a need of data concerning such resource utilization parameters within integrative pediatrics [45, 46]. Diagnoses-Related Groups (DRG), as well as length of stay are frequently used to inform health economic and resource utilization analyses in Germany, other European countries and worldwide [47–50]. Resource utilization analysis based on such data has rarely been used within integrative inpatient care [51, 52].

Therefore, the aim of the present study is to investigate parameters associated with resource utilization within integrative anthroposophic pediatric departments in Germany and to compare them systematically to representative data from all pediatric departments in Germany. Our hypotheses were that:

1. There is no difference considering the length of stay between integrative anthroposophic pediatric inpatient departments and a) the entirety of all pediatric departments in Germany, b) the DRG defined mean length of stay, as well the upper and lower limits.
2. Resource utilization indices, such as the effective Case Mix Index of integrative anthroposophic pediatric inpatient departments are comparable to the entirety of all pediatric departments in Germany.
3. There is no difference in the frequencies of DRG/ MDC between integrative anthroposophic pediatric departments and all pediatric departments in Germany.

## Methods

### Study design

The current study is a post hoc observational study. It was conducted according to the Declaration of Helsinki [53]. It is reported according to the STROBE guidelines for reporting observational cohort studies [54].

### Setting 

In Germany, there are two integrative hospitals focusing on Anthroposophic Medicine with pediatric inpatient departments: The Gemeinschaftskrankenhaus Herdecke (GKH) near Dortmund and the Filderklinik in Filderstadt near Stuttgart. Both hospitals treat children with various diseases reaching from general pediatrics to specialized fields by means of an integrative approach. This approach combines conventional and complementary remedies.

The pediatric department of the Filderklinik on average treats 1245 patients per year. Beneath general pediatrics, the Filderklinik specifies in neurology, psychosomatic disorders, neonatology, endocrinology, pulmonology and cardiology for children. In the pediatric ward of the GKH, 1750 patients are treated on average every year. The GKH practices diabetology, oncology, neonatology, rheumatology, psychosomatics and neurology in children alongside general pediatrics. The staff include physicians, nursing staff, pharmacists and therapists who are all trained in integrative medicine [43].

Diagnosis and treatment in both hospitals are in accordance with official pediatric guidelines from scientific societies and furthermore include treatment options from Anthroposophic Medicine [40, 55]. This anthroposophic treatment includes [43]: complementary pharmacotherapy, medicinal baths, rhythmical massages, compresses, and embrocation (rhythmic massages with etheric oils [38]), as well as art therapy, eurythmy, speech therapies, music therapy [39], and light/ color therapy [56]. Both hospitals are part of the German regular medical care and thus funded by the statutory health insurers.

### Data collection

Patient data over the last decade (2005–2016) was derived from the standard ward documentation interface Agfa-ORBIS® in all integrative anthroposophic pediatric departments. The Microsoft Excel®-output was imported into SPSS 24® (Statistical Package for the Social Sciences, IBM), cleaned and a plausibility check was performed. Furthermore, representative data was derived from the German National Consensus bureau for all pediatric departments in Germany (2005–2016).

### Eligibility criteria

There were no specific criteria of eligibility in the integrative anthroposophic sample. All patient cases of all integrative anthroposophic pediatric departments in Germany treated between 2005 and 2016 were included in the integrative sample. Outliers were excluded from analysis post hoc. An outlier is an observed value which deviates so much from the other values as to arouse suspicions that it was generated by a different mechanism [57]. In the data of the entirety of pediatric departments, outlier analysis was not possible since we were not able to gather raw data from the German consensus bureau. Consequently, exclusion of outliers was not possible.

### Sample

The integrative anthroposophic sample consists of 29,956 patient cases (Gemeinschaftskrankenhaus: *n* = 17,503 (58.4%); Filderklinik: *n* = 12,453 (41.6%). The sample of all pediatric departments in Germany includes 48,670,077 patient cases.

### Resource utilization parameters

In Germany, it is mandatory by law for all hospitals to provide data concerning health resource utilization to the Institute for the Hospital Renumeration System (InEK) and the National Consensus Bureau. These resource utilization parameters include Diagnosis Related Groups (DRG), Major Diagnosis Categories (MDC), and effective Case Mix Index (CMI). Therefore, these parameters are considered for comparisons between the integrative anthroposophic and all pediatric departments*.*

### Diagnosis related groups and major diagnosis categories

DRGs are assigned based on patients’ ICD-diagnosis, as well as procedures, age, sex, discharge status, and the presence of complications or comorbidities. In 2003 the G-DRG system was established in Germany [58] as an adaption of the Australian DRG system. It is updated annually by the Institute for the Hospital Remuneration System (InEK).

### Length of stay

The length of stay is measured in days in both samples. In the German DRG System only full days of stay are included for the length of stay [59]. Besides the length of stay, the German DRG system provides a mean length of stay, a minimum-, and a maximum length of stay for each diagnosis in the DRG-catalogue [59]. The length of stay of a patient can affect the revenue of a DRG. If the length of stay is shorter than the DRG defined lower limit, a deduction of the revenue is performed [60]. Vice versa, if the length of stay is longer than the upper limit, an additional fee is drawn [61]. For each DRG within the integrative anthroposophic sample the mean length of stay, as well as upper and lower limit for length of stay, were calculated using SPSS’ Syntax function.

The data source was the DRG case-based lump sum catalogues for the years 2005–2016 derived from the homepage of the Institute for the Hospital Remuneration System [62].

### Effective case mix index

In the German DRG-System (G-DRG), the cost weights are used to quantify a hospital’s average costs per case in relation to the specific resource utilization. This includes the Case-Mix (CM), which is equal to the sum of the cost weights of all DRGs performed over a given time period. The average case weight, which is called Case-Mix index (CMI), is calculated by dividing the CM by the total number of cases. Consequently, the CMI is equal to the average DRG cost weights for a particular hospital. The CMI is suitable for the comparison of the utilization of health care resources in different hospitals [63].

The effective CMI considers the deductions in the case of patient transfer or short-stay outliers, and surcharges for long-stay outliers and thus reflects the effort of a care provider for the treatment of a patient. An effective CMI value greater than 1.0 reflects a more extensive case compared to the average, while a value less than 1.0 indicates a less extensive case. In this way, the effective CMI maps the actual calculated amount for case fees [64]. Hence, in our study, the effective CMI of both samples were used for comparison of resource utilization between integrative anthroposophic and all German pediatric departments.

### Statistical analysis

All statistical analyses are performed using IBM SPSS Version 24 and R Statistics. Mean differences between the integrative anthroposophic sample and all pediatric departments are tested for statistical significance by means of t-tests for independent samples. Because of cumulative testing the level of statistical significance was Bonferroni adjusted to *p* < .01. Due to the high sample-size, Cohen’s d is calculated as a standardized measure of effect independent of the sample size.

## Results

### Length of stay

**Fig. 1:**
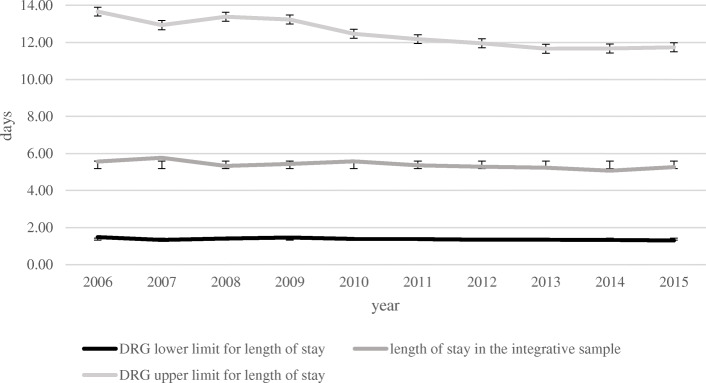
Mean length of stay in the integrative anthroposophic pediatric departments in comparison to upper and lower DRG defined length of stay limits

The mean length of stay in the integrative anthroposophic sample was 5.38 days (SD = 7.31, *n* = 29,956). Figure 1 illustrate the length of stay in the integrative sample compared to the DRG defined upper and lower limit of length of stay. The length of stay in the integrative anthroposophic sample did not exceed or undercut the DRG defined upper and lower limits for length of stay.

**Fig. 2:**
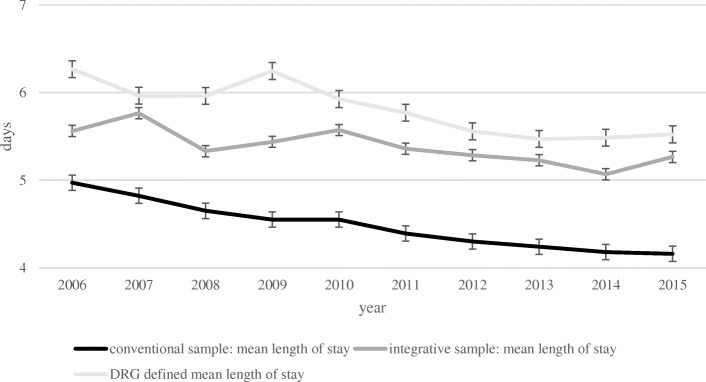
Mean length of stay in the integrative departments and all German pediatric departments compared to the DRG defined mean length of stay

Overall, the mean length of stay in the entirety of all pediatric departments was 4.48 days (SD = 7.83; *n* = 38,724,087). A t-test for independent samples showed a significant mean difference between the integrative anthroposophic and all pediatric departments (t (38,754,041) = 49.41 *p* < .01; Cohen’s d = 0.12). The average length of stay per year in the integrative anthroposophic and in all pediatric departments is shown in Table 1. The length of stay in the integrative anthroposophic sample was significantly lower (M = 4.74; SD = 6.23) than the mean length of stay defined by DRG (M = 5.8; SD = 4.71; t (28,236) = − 37.74; *p* < .01; Cohen’s d = − 0.07). The mean length of stay in the integrative anthroposophic and all pediatric departments compared to the mean length of stay proposed by DRG are shown in Fig. 2.

**Table 1:** Mean length of stay, CMI and Effective CMI per year in the integrative anthroposophic sample and in the conventional sample

	Mean length of stay		Effective Case Mix Index	
year	Integrative departments Mean (SD, n)	Entirety of all pediatric departments Mean (SD, n)	Integrative departments Mean (SD, n)	Entirety of all pediatric departments Mean (SD, n)
2006	5.56 (7.22; 2752)	4.97 (8.10; 841,100)	0.73 (1.10; 2826)	n.a.
2007	5.76 (6.96; 2792)	4.82 (7.88; 854,341)	0.71 (0.83; 2876)	n.a.
2008	5.33 (6.67; 2819)	4.65 (7.63; 859,058)	0.72 (1.18; 2903)	n.a.
2009	5.44 (7.20; 2841)	4.55 (7.84; 860,384)	0.80 (1.64; 2908)	n.a.
2010	5.57 (7.47; 2790)	4.55 (7.90; 860,961)	0.78 (1.33; 2859)	0.77 (1.99; 853,146)
2011	5.36 (7.23;2947)	4.39 (7.75; 866,611)	0.77 (1.32; 2992)	0.76 (1.98; 858,813)
2012	5.28 (7.53; 2947)	4.30 (7.55; 866,809)	0.74 (1.19; 3021)	0.75 (1.91; 858,809)
2013	5.23 (7.69; 3068)	4.24 (8.41; 879,100)	0.75 (1.21; 3138)	0.75 (2.00; 871,377)
2014	5.07 (7.01; 3228)	4.18 (7.67; 877,896)	0.77 (1.14; 3317)	0.76 (1.94; 870,090)
2015	5.27 (7.92; 3039)	4.16 (7.61; 878,913)	0.78 (1.17; 3116)	0.76 (1.99; 870,569)
overall	5.39 (7.29; 29,203)	4.48 (7.834; 8,645,173)	0.75 (1.23; 29,956)	0.76 (1.97; 5,182,804)

### Effective case mix index

The average effective CMI in the integrative anthroposophic sample is 0.76 (SD = 1.22; *n* = 29,956).

Overall the average effective CMI in the entirety of all pediatric departments was 0.76 (SD = 1.97; *n* = 39,159,515). The average effective CMI in the integrative and all German pediatric departments per year is shown in Table 1.

### Diagnoses related groups

The most frequent DRG in the integrative anthroposophic sample were B80Z (*head injuries*; *n* = 1933, 6.5%), G67B (*esophagitis, gastroenteritis, gastrointestinal bleeding, ulcer, complex genesis*; *n* = 1286, 4.3%), P67C (*newborn > 2499 g, without complex diagnosis*; *n* = 1254, 4.2%), G67C (*esophagitis, gastroenteritis, gastrointestinal bleeding, ulcer, uncomplex genesis*; *n* = 1158, 3.9%) and P67B (*n* = 975, 3.3%, *newborn > 2499 g, with complex diagnosis*).

In the entirety of all pediatric departments, the most frequent DRG’s were G67B (*esophagitis, gastroenteritis, gastrointestinal bleeding, ulcer, complex genesis*; *n* = 561,552; 8.78%) G67C (*esophagitis, gastroenteritis, gastrointestinal bleeding, ulcer, uncomplex genesis*; *n* = 440,529; 6.89%); B80Z (*head injuries*; *n* = 382,762; 5.99%) and D63Z (*otitis media or infections of the upper respiratory tract, age < 3 years; n* = 310,283; 4.85%). The 50 most frequent DRG in both groups per year and overall are shown in the supplemental materials 1 and 2.

### Major diagnosis categories

**Fig. 3:**
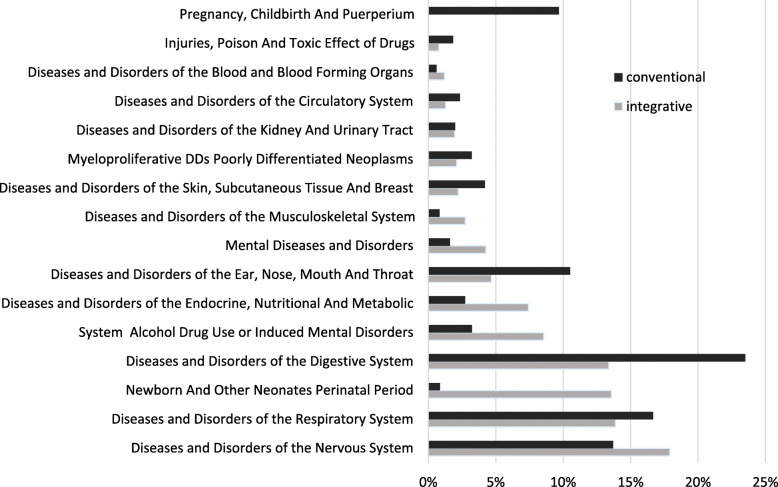
Percentages of MDCs in integrative anthroposophic departments and in the enterity of all German pediatric departments

The most frequent MDC in the integrative sample were *Diseases and Disorders of the Nervous System* (*n* = 5366, 17.90%), *Diseases and Disorders of the Respiratory System* (*n* = 4155, 13.87%), *Newborn and other Neonates Perinatal Period* (*n* = 4068; 13.58%) *and Diseases and Disorders of the Digestive System* (*n* = 4007; 13.38%). In the entirety of all pediatric departments in Germany sample the most frequent MDC were *Diseases and Disorders of the Digestive System* (*n* = 1,502,678; 23.50%); *Diseases and Disorders of the Respiratory System* (*n* = 1,066,127; 16.67%); *Diseases and Disorders of the Nervous System* (*n* = 876,894; 13.71%); *Diseases and Disorders of the Ear, Nose, Mouth and Throat* (*n* = 671,922; 10.51%). The percentages of the MDC compared in both samples are presented in Fig. 3.

There were some significant differences in the frequencies of the MDCs between the integrative pediatric departments and all German pediatric departments. Higher frequencies in the integrative sample were observed for the MDC: *Newborn and other Neonates Perinatal Period (*IPD: 13.88% vs. 0.87%); *Alcohol, Drug Use, Induced Mental Disorders* (IPD: 8.57 vs. 3.32%); *Mental Diseases and Disorders* (IPD: 4.27% vs. 1.16%); *Diseases and Disorders of the Endocrine, Nutritional and Metabolic System* (IPD: 7.43 vs 2.74); *Diseases and Disorders of the Nervous System* (IPD: 17.90% vs 13.71%).

Lower frequencies in the integrative sample were observed for the MDCs:

*Pregnancy, Childbirth and Puerperium* (IPD: 0.0% vs 9.6%); *Diseases and Disorders of the Digestive System* (IPD: 13.87% vs 23.05%); *Diseases and Disorders of Ear, Nose, Mouth and Throat* (IPD: 4.69% vs. 10.51%); *Diseases and Disorders of the Respiratory System* (IPD: 13.87% vs. 16.67%).

## Discussion

In this study, we aimed to investigate resource utilization parameters of integrative anthroposophic pediatric departments and to compare them to corresponding data from all pediatric departments in Germany. In accordance with our initial hypothesis, we found no difference between pediatric integrative anthroposophic departments and the entirety of all pediatric departments concerning effective Case Mix Index. The length of stay in the integrative departments was shorter than the mean DRG-defined mean length of stay and within upper and lower limits, which was in line with our hypothesis.

Furthermore, we hypothesized that these department do not differ from the entirety considering patients’ length of stay. Contrary to this hypothesis, we found that the mean length of stay was significantly longer in the integrative anthroposophic departments compared to all German pediatric departments. Another hypothesis was that the departments do not differ considering the frequency distribution of DRG and MDC. Our data did not support this hypothesis, but much more implied some systematic discrepancies between the integrative anthroposophic pediatric departments and all German pediatric departments.

### Length of stay

The average length of stay in the integrative sample was significantly lower than the mean length of stay defined by DRG. It did furthermore, not exceed the upper limit of length of stay defined by DRG or undercut the lower limit of length of stay defined by DRG. This result implies that integrative pediatric departments in Germany can provide care within the terms of the DRG defined conditions concerning length of stay. In contrast to previous studies, we found no indication for less resource utilization in the integrative departments [65, 66].

The mean length of stay in the integrative anthroposophic departments was significantly longer compared to the mean stay in all German pediatric departments. This finding is in line with previous research that found longer length of stay in integrative anthroposophic [51] and integrative naturopathic departments [45]. This circumstance may most likely be due to the large number of time-consuming diagnostic and medical procedures that are associated with integrative anthroposophic treatment. Previous studies found an association of increased length of stay in integrative medical department with the utilization of additional anthroposophic [51] or naturopathic [45] reimbursement, which requires a longer stay.

Considering this, the relative difference of 1 day in the length of stay between the departments is comparatively low. While this difference is statistically significant, the effect size is low. However, in this context, it needs to be stated that outliers with extreme lengths of stay (mainly from the diagnosis spectrum of eating disorders) were excluded prior to analysis.

### Effective case mix index

The mean effective CMI were identical in the integrative sample and in all German pediatric departments. This finding indicates that integrative pediatric departments have comparable resource utilization management to general pediatric departments, which is in line with comparable cost analyses [45].

### Diagnosis related groups and major diagnosis categories

In both samples, *esophagitis, gastroenteritis, gastrointestinal bleeding, ulcer (G67B; G67C) and head injuries* (B80Z) belonged to the most frequent DRG’s. The percentages varied between the integrative anthroposophic and all pediatric departments. While *newborn > 2499 g, without complex diagnosis* was one of the most frequent DRG’s in the integrative anthroposophic sample, *otitis media or infections of the upper respiratory tract age < 3 years* (D63Z) was more frequent in the entirety of all German pediatric departments.

The frequencies of the MDCs in the integrative anthroposophic sample showed some significant differences in comparison to the entirety of all pediatric departments in Germany. Higher frequencies could be obtained for MDCs of chronic diagnosis spectrum, such as mental, endocrine, and nervous disorders. Lower frequencies were found for acute diseases, such as digestive, respiratory, and ENT- disorders. A similar pattern was obtained in the DRG frequencies.

This result pattern is known from a previous study of our working group on the patient characteristics and clinical characteristics of integrative anthroposophic pediatric departments in Germany [43]. Furthermore, this result is in line with other international studies that conclude that the use of integrative medicine seems to be more frequent in in children with severe and chronic diseases [7, 16, 30–36]. The higher frequency of chronic and severe diseases may be another factor influencing the longer length of stay in the integrative pediatric departments.

The large difference considering the MDC newborns, neonates and diseases of the perinatal period, is most likely due to the specification of the GKH with its center for neonatology. The absence of pregnancy, childbirth, and puerperium in the integrative anthroposophic sample may be explained by the circumstance that the treatment in this MDC is merely used by the gynecologic department in the integrative anthroposophic hospitals but not by the pediatric department. The higher percentage of this MDC in all German pediatric departments may be caused by teenage pregnancies or mothers who are treated in the pediatric department because their neonate child is treated in the pediatric department.

### Strengths and limitations

The aim of the present study was to contribute to the better understanding of resource utilization, as measured by length of stay, in pediatric integrative medicine in Germany. A big strength of this study is that it is the first systematic investigation of a large sample of integrative pediatric resource utilization data with comparison to representative data of the entirety of all pediatric inpatient departments in Germany. One major limitation of this study is that it is a secondary data analysis. We were not able to gain raw data from the German Federal Statistical Office for the entirety of pediatric hospitals in Germany. Consequently, it was not possible to exclude any outliers in this sample. Future analyses also need to look at the impact on resource utilization in primary and outpatient care, as well as rehabilitation and social care where appropriate as they may influence the length of stay of in-patients.

We also recognize that this analysis only provides one aspect of information required for future economic evaluation; sequential services and the use of resources for the entire episode of care were not addressed in this study. To do so, data on resource use and their costs between integrated pediatric hospitals and other pediatric hospitals will need to be combined with comparative data on outcomes associated with treatment in these settings. This would include analysis for different population sub-groups, for instance by different MDC. Ideally outcomes would be measured in terms of impact on quality of life so that the health economic gold standard of incremental cost per quality adjusted life year (QALY) gained could then be assessed. It would also be important to look at whether there are differences in patterns of rehospitalization as part of any future economic evaluation.

## Conclusions

The comparison of resource utilization in integrative anthroposophic pediatric departments to the entirety of pediatric departments in Germany shows a heterogeneous pattern of similarities and differences. The effective Case Mix Indices were identical, indicating an equal resource utilization in integrative anthroposophic and all pediatric departments. Treatment within integrative anthroposophic pediatric departments fits well in terms of the DRG defined conditions concerning length of stay, even though integrative pediatric patients has an increased length of stay of averagely 1 day, which is most likely associated to time consuming, complex integrative treatment approaches and to a certain extend to higher amount of chronic and severe diseases. Future economic evaluations are needed to assess whether integrative anthroposophic pediatric departments is cost effective.

## Supplementary information

**Additional file 1 Supplemental material 1**: 50 most frequent DRG in intergative anthroposophic departments in Germany.

**Additional file 2 Supplemental material 2**: 50 most frequent DRGs in the entirety of all pediatric departsments in Germany.

## Data Availability

Raw data are available only for analysis purposes and only to dedicated staff of our research group. As the original data is patient hospital data we have no permission to share it.

## Abbreviations

IPD: Integrative Pediatric Departments
CMI: Effective Case Mix Index
DRG: Diagnoses Related Groups
GKH: Gemeinschaftskrankenhaus Herdecke
InEK: Institute for the Hospital Remuneration System
M: Mean
Max: Maximum
MDC: Major Diagnostic Category
Min: Minimum
SD: Standard Deviation
SPSS: Statistical Package for the Social Sciences
STROBE: Strengthening the Reporting of Observational Studies in Epidemiology
